# Supplementary material for: Quantum channel correction outperforming direct transmission
Source: Nat Commun. 2022 Apr 5;13:1832. doi: 10.1038/s41467-022-29376-4 (PMC8983674; doi:10.1038/s41467-022-29376-4)
Supplement: Supplementary file 1 — Supplementary Information [file 41467_2022_29376_MOESM1_ESM.pdf]

Supplementary Information
for
Quantum channel correction outperforming direct transmission

Sergei Slussarenko,[1,*] Morgan M. Weston,[1] Lynden K. Shalm,[2] Varun B. Verma,[2] Sae-Woo Nam,[2] Sacha Kocsis,[1,3] Timothy C. Ralph,[4,†] and Geoff J. Pryde[1,‡]

[1]*Centre for Quantum Dynamics and Centre for Quantum Computation and Communication Technology, Griffith University, Brisbane, Queensland 4111, Australia*
[2]*National Institute of Standards and Technology, 325 Broadway, Boulder, Colorado 80305, USA.*
[3]*Current address: Centre for Quantum Computation and Communication Technology, The University of New South Wales, Sydney 2052, Australia*
[4]*Centre for Quantum Computation and Communication Technology, School of Mathematics and Physics, University of Queensland, St Lucia, Queensland 4072, Australia*

CONTENTS

Supplementary Section 1: Source data 2
Supplementary Section 2: Heralded amplification background theory 2
Supplementary Section 3: Protocol rates data and discussion 3

LIST OF TABLES

I Concurrence measurements C_{hv} of entanglement distributed via the error-corrected quantum channel 2
II Absolute values of the density matrix elements of the state ρ_{fe} 2
III Absolute values of the density matrix elements of the state ρ_{hv} 2
IV Error-corrected channel preparation rate 4
V Error-corrected channel state preparation and sending rates 4

LIST OF FIGURES

1 Hong-Ou-Mandel interference at HA and ES stages 4
2 Logarithmic plot of the operating rates of the error-corrected and direct transmission quantum channels 5

* s.slussarenko@griffith.edu.au
† ralph@physics.uq.edu.au
‡ g.pryde@griffith.edu.au

Supplementary Section 1: Source data

Supplementary Table I | Concurrence measurements C_{hv} of entanglement distributed via the error-corrected quantum channel as a function of η. **a** Data for the case of $L = 0.9884 \pm 0.0006$, **b** Data for the case of $L = 0.958 \pm 0.001$, **c** Data for the case of $L = 0.903 \pm 0.002$. Reported uncertainties ΔC correspond to the experimentally observed statistical uncertainty of ± 1 standard deviation. Green (red) background highlights the cases when the observed concurrence was higher (lower) that the concurrence C_{fe} of the state distributed directly through loss, taking into account the uncertainties. $C_{fe} = 0.065 \pm 0.001$,0.121 ± 0.002, and 0.184 ± 0.002 for **a**, **b**, and **c**, respectively. For the gain setting $\eta = 0.0049$ and loss $L = 0.9884$ (i.e. the results highlighted in bold in **a**), the concurrence is estimated theoretically to be increased to ≈ 0.52, under the assumptions of perfect HOM interference, a fully-lossless setup (other than loss L on the channel), unit-efficiency threshold detection, but taking into account high-order photon number noise. The expected direct transmission concurrence for the same experimental conditions is estimated to be ≈ 0.1.

a $L = 0.9884 \pm 0.0006$

η	C_{hv}	ΔC_{hv}
0.0012	0.22	0.03
**0.0049**	**0.27**	**0.02**
0.0076	0.23	0.02
0.0302	0.14	0.02
0.0670	0.08	0.01
0.1170	0.06	0.01
0.1786	0.033	0.006
0.2500	0.022	0.007

b $L = 0.958 \pm 0.001$

η	C_{hv}	ΔC_{hv}
0.0012	0.13	0.03
0.0049	0.21	0.03
0.0076	0.23	0.04
0.0302	0.23	0.01
0.0670	0.16	0.02
0.1170	0.13	0.01
0.1786	0.09	0.01
0.2500	0.08	0.01

c $L = 0.903 \pm 0.002$

η	C_{hv}	ΔC_{hv}
0.0012	0.10	0.03
0.0049	0.17	0.03
0.0076	0.22	0.04
0.0302	0.25	0.01
0.0670	0.22	0.02
0.1170	0.17	0.01
0.1786	0.13	0.01
0.2500	0.12	0.01

Supplementary Table II | Absolute values of the density matrix elements (Fig. 4a, c, e) of the state ρ_{fe} transmitted directly through loss. **a** Data for the case of $L = 0.9884 \pm 0.0006$, **b** Data for the case of $L = 0.958 \pm 0.001$, **c** Data for the case of $L = 0.903 \pm 0.002$.

a $L = 0.9884 \pm 0.0006$

	$\langle 0,0\|$	$\langle 1,0\|$	$\langle 0,1\|$	$\langle 1,1\|$
$\|0,0\rangle$	0.67376	0	0	0
$\|1,0\rangle$	0	0.32113	0.04016	0
$\|0,1\rangle$	0	0.04016	0.00502	0
$\|1,1\rangle$	0	0	0	0.00008

b $L = 0.958 \pm 0.001$

	$\langle 0,0\|$	$\langle 1,0\|$	$\langle 0,1\|$	$\langle 1,1\|$
$\|0,0\rangle$	0.68105	0	0	0
$\|1,0\rangle$	0	0.30296	0.06935	0
$\|0,1\rangle$	0	0.06935	0.01587	0
$\|1,1\rangle$	0	0	0	0.00011

c $L = 0.903 \pm 0.002$

	$\langle 0,0\|$	$\langle 1,0\|$	$\langle 0,1\|$	$\langle 1,1\|$
$\|0,0\rangle$	0.66403	0	0	0
$\|1,0\rangle$	0	0.30447	0.09784	0
$\|0,1\rangle$	0	0.09784	0.03144	0
$\|1,1\rangle$	0	0	0	0.00005

Supplementary Table III | Absolute values of the density matrix elements (Fig. 4b, d, f) of the state ρ_{hv} transmitted through the error corrected channel. **a** Data for the case of $L = 0.9884 \pm 0.0006$, **b** Data for the case of $L = 0.958 \pm 0.001$, **c** Data for the case of $L = 0.903 \pm 0.002$.

a $L = 0.9884 \pm 0.0006$

	$\langle 0,0\|$	$\langle 1,0\|$	$\langle 0,1\|$	$\langle 1,1\|$
$\|0,0\rangle$	0.67284	0	0	0
$\|1,0\rangle$	0	0.11722	0.13349	0
$\|0,1\rangle$	0	0.13349	0.20994	0
$\|1,1\rangle$	0	0	0	0

b $L = 0.958 \pm 0.001$

	$\langle 0,0\|$	$\langle 1,0\|$	$\langle 0,1\|$	$\langle 1,1\|$
$\|0,0\rangle$	0.52014	0	0	0
$\|1,0\rangle$	0	0.06537	0.13310	0
$\|0,1\rangle$	0	0.13310	0.41401	0
$\|1,1\rangle$	0	0	0	0.00049

c $L = 0.903 \pm 0.002$

	$\langle 0,0\|$	$\langle 1,0\|$	$\langle 0,1\|$	$\langle 1,1\|$
$\|0,0\rangle$	0.58178	0	0	0
$\|1,0\rangle$	0	0.08891	0.14157	0
$\|0,1\rangle$	0	0.14157	0.32895	0
$\|1,1\rangle$	0	0	0	0.00037

Supplementary Section 2: Heralded amplification background theory

The effect of loss on a single rail qubit is equivalent to an amplitude damping channel. In particular the pure initial state $\alpha|0\rangle + \beta|1\rangle$ is taken to:

$$|\beta|^2(1-T)|0\rangle\langle 0| + (\alpha|0\rangle + \sqrt{T}\beta|1\rangle)(\alpha^*\langle 0| + \sqrt{T}\beta^*\langle 1|) \tag{S1}$$

The successful action of the NLA with gain g on this state gives:

$$\frac{|\beta|^2(1-T)|0\rangle\langle 0| + (\alpha|0\rangle + g\sqrt{T}\beta|1\rangle)(\alpha^*\langle 0| + g\sqrt{T}\beta^*\langle 1|)}{1 + T|\beta|^2(g^2-1)} \tag{S2}$$

The fidelity between the initial and final states is:

$$F = \frac{|\beta|^2(1-T)(1-|\beta|^2) + (1-|\beta|^2 + g\sqrt{T}|\beta|^2)^2}{1 + T|\beta|^2(g^2-1)} \tag{S3}$$

The fidelity is improved by successful action of the NLA by choosing an optimal value of the gain. However, the optimal gain is state dependent (and T dependent). To remove the state dependence we can consider the average fidelity over the Block sphere, given by:

$$F_{av} = \int_0^1 F d|\beta|^2 \tag{S4}$$

which still shows an advantage from use of the NLA with an optimized gain. The disadvantages of this approach are that the improvement is limited and the state is destroyed when the NLA doesn't succeed.

An alternative approach is to send one arm of the entangled state $\sqrt{\varepsilon}|01\rangle + \sqrt{1-\varepsilon}|10\rangle$ through the channel. After the loss and successful application of the NLA with the gain $g\sqrt{\varepsilon T} = \sqrt{1-\varepsilon}$ the state is:

$$\frac{\varepsilon(1-T)|00\rangle\langle 00| + (1-\varepsilon)(|01\rangle + |10\rangle)(\langle 01| + \langle 10|)}{2(1-\varepsilon) + (1-T)\varepsilon} \tag{S5}$$

In the limit that ε is small one approximately retrieves the maximally entangled state $\frac{1}{\sqrt{2}}((|01\rangle + |10\rangle)$. In principle this state can be then be used to teleport arbitrary states forming an effective identity channel in spite of arbitrary loss on the physical channel. In practice the feedforward gain becomes very large in this limit and hence the probability of preparing the state becomes very low making this regime difficult to achieve. In the experiment we use equal superposition states ($\varepsilon = 1/2$) and find that for a range of gains we are able to demonstrate error corrected channels which outperform the direct channel by a large margin.

Supplementary Section 3: Protocol rates data and discussion

A fair comparison between the direct transmission and error-corrected channel performance requires precise accounting of the operation rates of the two channels. Due to the probabilistic nature of SPDC, the probability of generating two photon pairs for the full protocol is lower than the probability of generating a single pair for the direct transmission. This, together with the fact that HA success rate decreases with the increase of gain setting, leads to the lower absolute counts per second for the error corrected channel compared to the direct transmission.

In our analysis, however, we use a more relevant definition of the operation rate as the rate at which input states are prepared and sent through the channel, given successful preparation of the channel. For the error-corrected channel, the preparation of the channel is heralded by the joint heralding signal from HA stage and the herald of the ancilla photon. The success of the input state preparation and transmission through the channel is heralded by the joint signal from the ES, HA and the ancilla herald detection. The corresponding experimental heralding rates are shown in Supplementary Tables IV and V for all three values of added loss and different gain settings of the HA. The operation rate of the direct transmission channel is defined as the ratio between the the rate of state heralding and the pulse rate of the laser. Supplementary Figure 2 shows the comparison between operation rates for the two types of channels.

For low amplifier gain and low amplitude of the state input to HA, the probability of success of the amplifier is approximately independent of the input state. In this regime the firing of the entanglement swapping detectors and the noiseless amplification detectors are independent events and we see the true rate of entanglement swapping normalized to the random rate of noiseless amplification successes. In this regime we see equal rates for direct transmission and error-corrected transmission. The use of photon number resolving detectors at the entanglement swapping stage would lead to the 50% failure rate of the teleported channel, which would need to be taken into account for the correct comparison of the concurrences. Instead, the use of threshold detectors, although decreasing the final concurrence of the state sent through the error-corrected channel, provides a level playing field for the comparison of the two scenarios.

When the gain of the amplification is high, i.e. $\eta \approx 0$, the probability of the HA resrource state $|\psi_{\mathrm{av}}\rangle = \sqrt{\eta}|1_a 0_v\rangle + \sqrt{1-\eta}|0_a 1_v\rangle$ to be transmitted inside HA stage becomes comparable with the probability of receiving the entangled resource state $|\psi_{\mathrm{fe}}\rangle$. The success of amplification thus becomes affected by the amplitude of $|\psi_{\mathrm{fe}}\rangle$ sent through the channel. As the preparation of the input state $|\psi_{\mathrm{hg}}\rangle$ and the preparation of the entangled state $|\psi_{\mathrm{fe}}\rangle$ are a correlated event for our particular setup, the success of the swapping and the amplification become correlated too. This leads to the rate in the case of the error corrected channel being higher than direct transmission. However, given this is a peculiarity of our specific set-up, we consider the true rate to be that determined in the low gain limit. Given then equal rates, the direct comparison of the concurrences in the two cases is justified.

Supplementary Table IV | Error-corrected channel preparation rate. The rates of heralding signal from HA stage, measured in Hz, as a function of η, for three different values of added loss L.

η	0.0012	0.0049	0.0076	0.0302	0.0670	0.1170	0.1786	0.2500
$L = 0.9884 \pm 0.0006$	76.75 ± 0.07	165.6 ± 0.1	242.3 ± 0.1	988.9 ± 0.6	1996.1 ± 0.3	3587.6 ± 0.5	4411.0 ± 0.5	7303.1 ± 0.8
$L = 0.958 \pm 0.001$	50.22 ± 0.05	113.82 ± 0.09	160.4 ± 0.1	580.4 ± 0.2	1329.3 ± 0.3	2540.1 ± 0.6	4026.8 ± 0.7	5654.9 ± 0.8
$L = 0.903 \pm 0.002$	49.40 ± 0.06	85.42 ± 0.08	150.9 ± 0.1	478.3 ± 0.2	1243.2 ± 0.4	2542.1 ± 0.5	3969.7 ± 0.6	5604.0 ± 0.8

Supplementary Table V | Error-corrected channel state preparation and sending rates. The rates of joint heralding signal from ES and HA stages, measured in Hz, as a function of η, for three different values of added loss L.

η	0.0012	0.0049	0.0076	0.0302	0.0670	0.1170	0.1786	0.2500
$L = 0.9884 \pm 0.0006$	0.062 ± 0.002	0.099 ± 0.002	0.127 ± 0.003	0.43 ± 0.01	0.824 ± 0.007	1.47 ± 0.01	1.77 ± 0.01	2.99 ± 0.02
$L = 0.958 \pm 0.001$	0.101 ± 0.002	0.117 ± 0.003	0.133 ± 0.003	0.281 ± 0.005	0.524 ± 0.007	0.94 ± 0.01	1.47 ± 0.01	2.00 ± 0.01
$L = 0.903 \pm 0.002$	0.183 ± 0.004	0.158 ± 0.004	0.207 ± 0.005	0.282 ± 0.005	0.499 ± 0.007	1.03 ± 0.01	1.50 ± 0.01	2.04 ± 0.01

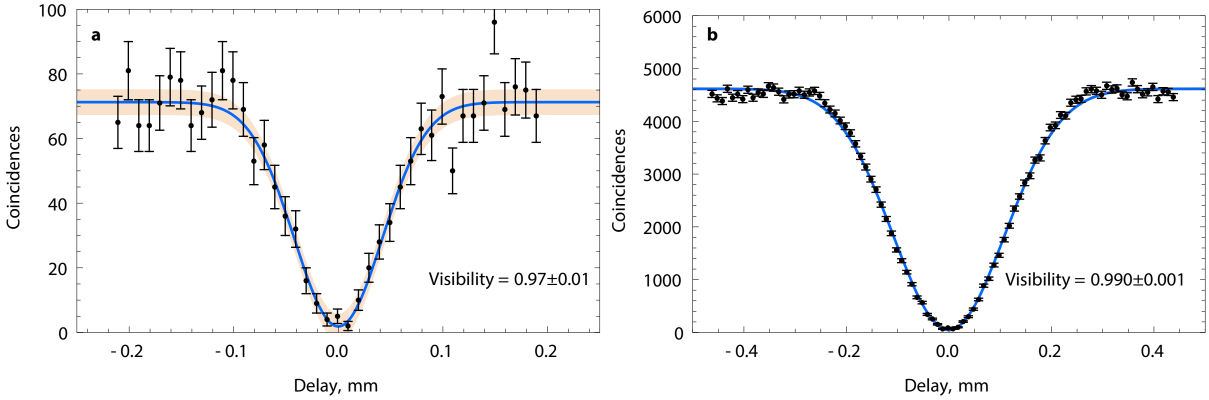

Supplementary Figure 1 | Hong-Ou-Mandel interference at HA and ES stages. a Four-photon coincidence rate of a HOM interference at the HA stage as a function of an optical delay introduced between the interfering photons. Mild, $\approx$ 8 nm FWHM, spectral filtering applied on the herald photons only. **b** Two-photon coincidence rate of a HOM interference at the ES stage. Same $\approx$ 8 nm FWHM spectral filtering is applied to interfering photons, resulting only in the widening of the HOM dip, with minimal effect on interference visibility. Error bars correspond to the experimentally observed statistical uncertainty of ± 1 standard deviation. Shaded areas correspond to the 95% confidence region, derived from uncertainty in the fit parameters.

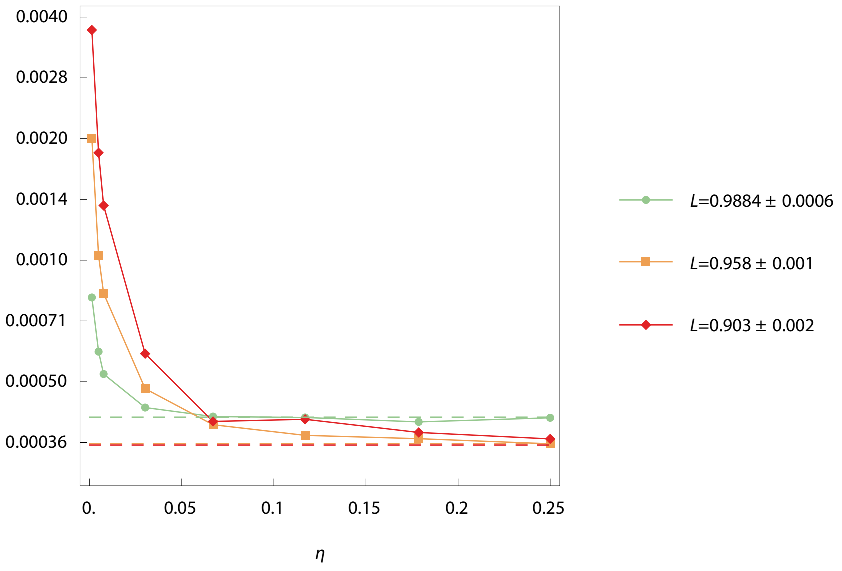

Supplementary Figure 2 | Logarithmic plot of the operating rates of the error-corrected and direct transmission quantum channels for three different values of added loss. Dashed lines represent the experimentally measured operating rate of the direct transmission through loss, and dots represent the operating rates of the error-corrected channel as a function of the HA gain setting. Error bars are smaller than the dots size.
